# Surveillance of respiratory syncytial virus infections in adults, Austria, 2017 to 2019

**DOI:** 10.1038/s41598-021-88537-5

**Published:** 2021-04-26

**Authors:** Lorenz Schubert, Johanna Steininger, Felix Lötsch, Anna Nele Herdina, Monika Redlberger-Fritz, Selma Tobudic, Michael Kundi, Robert Strassl, Christoph Steininger

**Affiliations:** 1 Division of Infectious Diseases and Tropical Medicine, Department of Medicine I, Medical University of Vienna, Waehringer Guertel 18-10, 1090 Vienna, Austria; 2 Division of Clinical Virology, Department of Laboratory Medicine, Medical University of Vienna, Vienna, Austria; 3 Department of Virology, Medical University Vienna, Vienna, Austria; 4 Department for Environmental Health, Center for Public Health, Medical University of Vienna, Vienna, Austria

**Keywords:** Viral infection, Preventive medicine, Epidemiology

## Abstract

Respiratory syncytial virus (RSV) testing is generally available in most care centres, but it is rarely performed because clinicians’ seldom suspect RSV to be the underlying pathogen in adults with respiratory disease. Here, we evaluate the impact of broad combined influenza/RSV testing on the clinical practice. Overall, 103 patients were tested positively for RSV. Our study indicates that positively tested patients were mostly of advanced age and suffered from chronic diseases. Mortality was significant in our cohort and higher in patients with advanced age. Further, we report a significant increase in detected RSV cases but also in detection rate. Together, these findings suggest that implementation of a combined influenza/RSV testing led to a significant increase in detection rate, supported clinicians establishing the correct diagnosis and allowed a safe and controlled handling of RSV patients.

## Introduction

The respiratory syncytial virus (RSV), a single-stranded RNA virus, is primarily known as a major cause of lower respiratory tract infection in infants and young children^1^. In the recent past, numerous studies have demonstrated significant disease burden in adults, especially if immunocompromised, suffering from an oncological disease, chronic obstructive pulmonary disease, severe asthma or congestive heart failure^2,3^. Shi et al. estimated a global number of 336,000 hospitalized patients per year due to RSV in the group of older adults (≥ 65 years)^4^.

Previously, an overwhelming amount of studies reported similar rates of hospitalization and mortality of influenza virus A and B compared to RSV^5–8^. Similar clinical presentation of both viruses and comparable seasonal characteristics makes a differential diagnosis based on clinical or epidemiological characteristics nearly impossible without adequate diagnostics^9^. Moreover, both viruses can be transmitted by aerosol droplets or direct contact to the virus, such as over fomites^10,11^. Hence, prevention protocols have to be implemented to protect health care workers (HCWs) and prevent disease transmission within health care facilities.

Despite overwhelming evidence that RSV is associated with a significant disease burden in adults, clinicians’ awareness is still low^12,13^. At the Vienna General Hospital, 2776 samples were tested for Influenza A and B, however only 387 samples were tested for RSV during season 2017/2018. In autumn 2018, a combined flu/RSV test was introduced at the Vienna General Hospital. Hence, patients with suspected respiratory tract infection tested for influenza were automatically tested for RSV and treated according to strict infection control and prevention strategies (supplementary Table 1).

Here, we set out to describe RSV disease burden in hospitalized adults at a university hospital, highlight the impact on the clinical practice and elucidate pitfalls of misinterpretation with other respiratory viruses.

## Methods

This retrospective cohort analysis assesses the influence of a newly implemented combined influenza/RSV RT-PCR test on the clinical practice at a university hospital. The study protocol was approved by the Ethics Committee of the Medical University of Vienna, Austria (ECS 1523/2019) and all study-related procedures were conducted according to the declaration of Helsinki. This manuscript is reported following the RECORD statement^14^.

### Data collection and analysis

**Figure 1:**
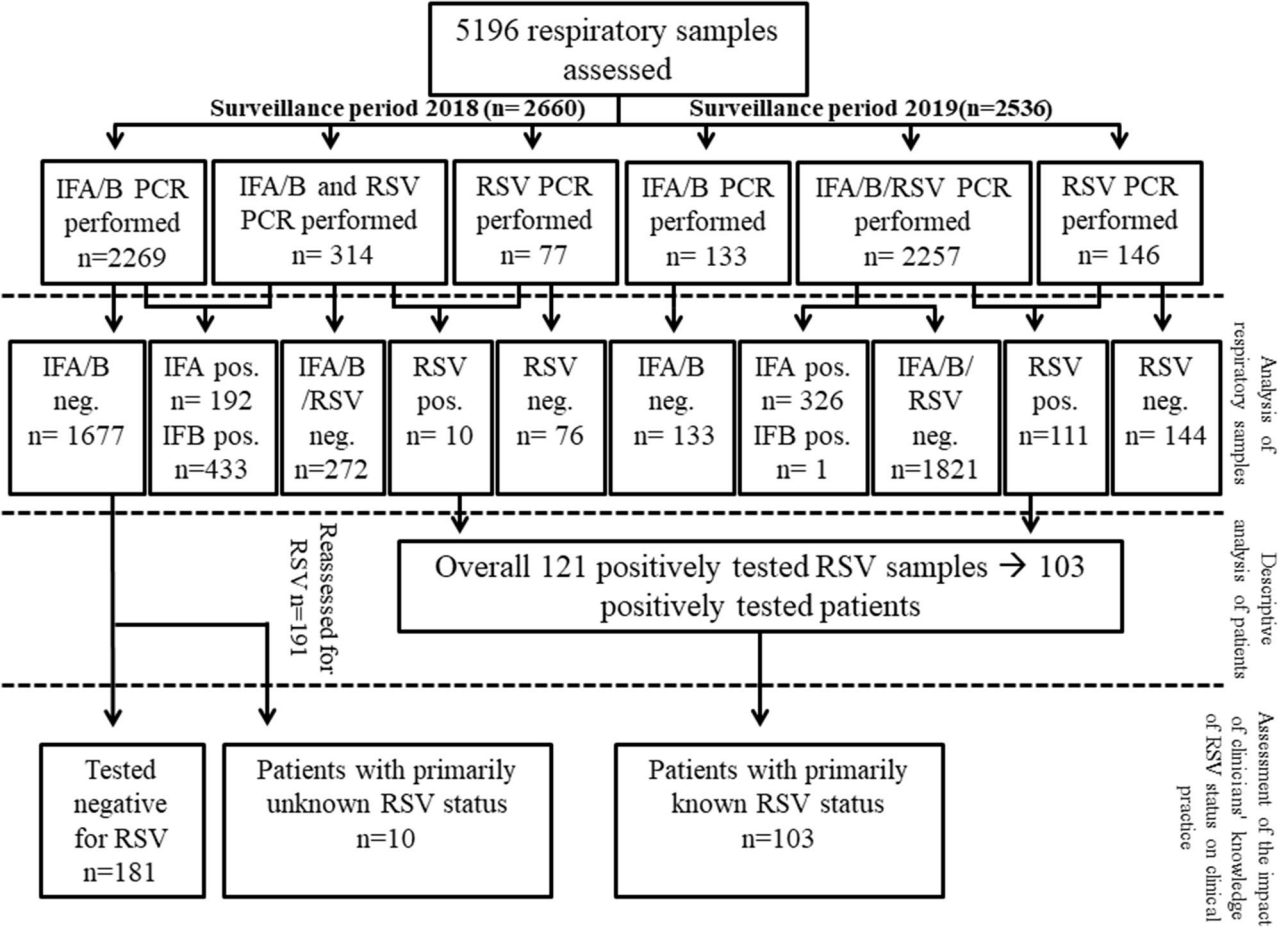
Demonstrates the flow-chart of included samples and performed analysis; IFA, influenza A; IFB, influenza B; RSV, respiratory syncytial virus.

Firstly, all samples of patients with suspected influence like illness admitted to the General Hospital during October 2017 to April 2019 were included. Detection rate of viruses (RSV, influenza A and influenza B) were described in dependence of the surveillance period. Secondly, all RSV positively tested patients during October 2017 to April 2019 were included into the data analysis. Figure 1 demonstrates the investigated collective and performed analysis. For the data analysis demographic parameters (age, sex, body-mass index, co-morbidities and history of smoking), microbiological data (urinary antigen testing, blood-culture, bronchoalveolar lavage fluid culture), radiological data (evidence of pneumonia), laboratory parameters (haemoglobin, thrombocytes, leucocytes, creatinine, bilirubin and C-reactive protein), treatment procedures (admission to hospital, administration of antibiotics, admission to ICU) and mortality data were retrospectively extracted from the electronic patient information system. Firstly, diagnostic approach of RSV was compared between the surveillance period 2018 (period before combined influenza/RSV testing), defined as week 40 of 2017 to week 30 of 2018, and the surveillance period 2019 (period with influenza/RSV testing in place), defined as week 40 of 2018 to week 30 of 2019. Secondly, demographic data, handling of the patients and outcome were described. Thirdly, differences of the handling of RSV patients were assessed in dependence of clinicians’ knowledge of the RSV status (RSV status known versus RSV status not known). Therefore, an additional cohort was formed by retrospectively assessing 191 sputum samples of patients admitted to the hospital due to respiratory symptoms during the encompassed peak of 2018 RSV season in Vienna (week 8 to 12) but not tested for RSV infection, for RSV infection. RSV peak was defined in accordance to RSV peak published by Austria’s Sentinel Physician Surveillance Network^15,16^.

### Diagnostic assays performed

Tests were performed differently depending on the season. (1) in season 2018 testing for influenza virus A and B was performed by RT-PCR (in-house protocol established after Ward et al. or Xpert Xpress Flu, Cepheid) and testing for RSV was performed by (MagNA Pure 96 system, TaqMan-based RT-PCR using the LightCycler Multiplex RNA Virus Master [Roche Diagnostics, Rotkreuz, Switzerland] on a Roche Lightcycler 480II thermocycler [Roche Diagnostics, Rotkreuz, Switzerland]^17^. RT-PCR was performed using the routine test for respiratory viruses in-house established after Fry et al., but not always in parallel^18^. (2) in season 2019 RSV infection was diagnosed by a combined Flu/RSV RT-PCR (Xpert Xpress Flu/RSV XC, Cepheid). (3) 191 samples, not tested for RSV infection during the encompassed peak of 2018 RSV season in Vienna (week 8 to 12), were reassessed for RSV by RT-PCR with the above mentioned routine protocol.

### Statistical analysis

Statistical analysis was performed using commercially available computer software SPSS Statistics 20 (IBM, USA) and figures were produced in R (R Core Team, 2014) using the package ggplot2 (Wickham 2016)^19,20^.

Baseline characteristics of participants are summarized as frequencies and proportions for categorical data and as means and standard deviations or medians and interquartile for metric data. The hypothesis testing was performed by Chi-Square test for categorical variables and Student’s t tests or Mann–Whitney tests for metric data. To illustrate correlations between categorical variables, a Pearson-correlation is performed for normally distributed variables, as well as Spearman's Rho for non-parametric variables. Statistical significance was defined as p < 0.05.

### Ethics declaration

The study protocol of this retrospective analysis was approved by the Ethics Committee of the Medical University of Vienna, Austria (ECS 1523/2019) and all study-related procedures were conducted according to the declaration of Helsinki. Due to the retrospective study design, informed consent was waived by the Ethics Committee of the Medical University Vienna.

## Results

### Analysis of respiratory samples collected during season 2018 and 2019

**Figure 2:**
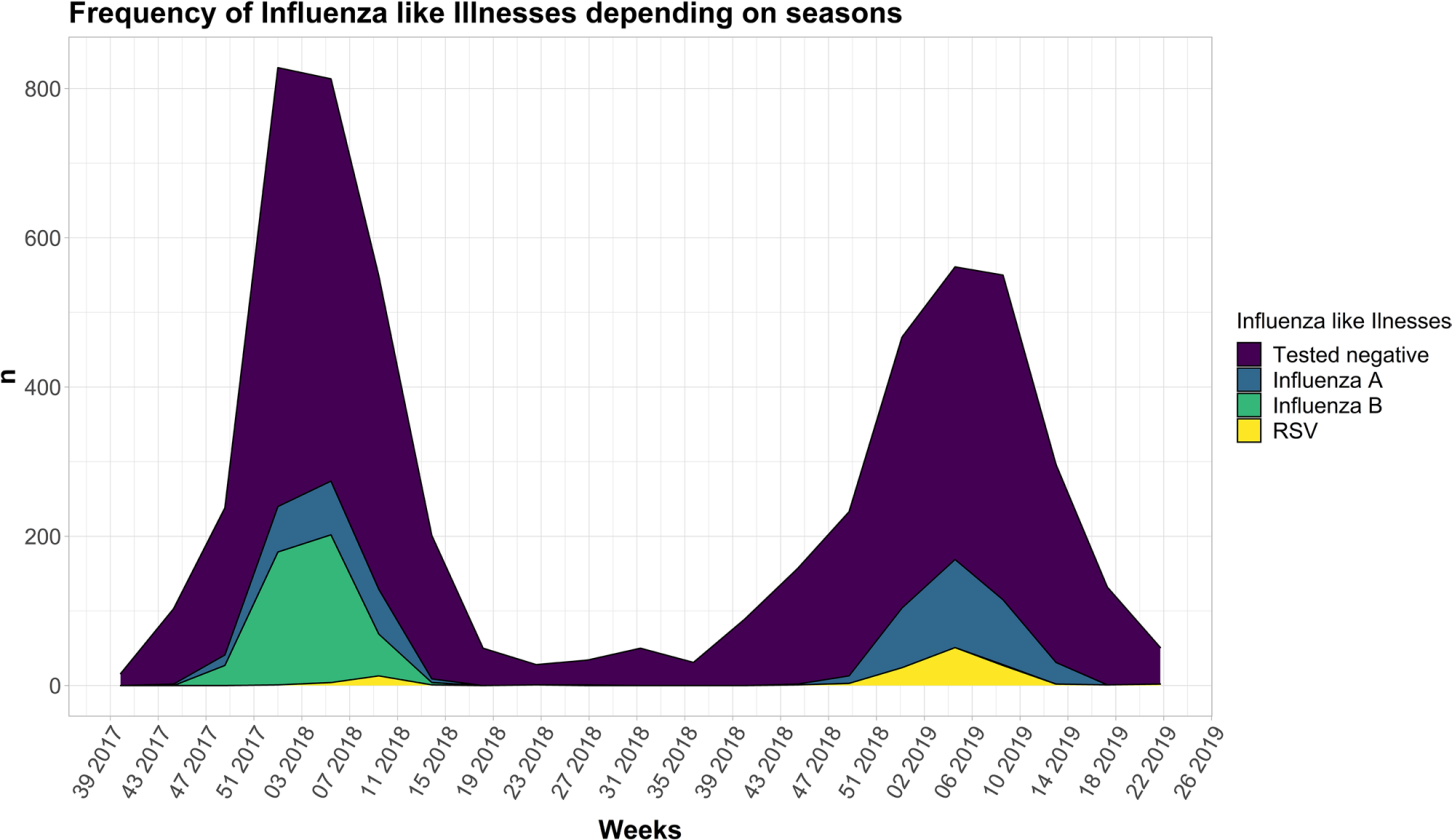
Demonstrates the frequency of influenza like illnesses depending on the seasons^20^.

A total 5196 respiratory samples were collected, 2660 samples during season 2018 and 2536 samples during season 2019. Performed tests and results are demonstrated in Fig. 2. Interestingly, there was a significant difference in detection rate between the seasons for all of the reported viruses. We detected a significant increase (p < 0.001) in influenza A in season 2019 (326/2390 [13.6%]) compared to season 2018 (192/2583 [7.4%]). Prevalence of influenza B was significantly lower (p < 0.001) in season 2019 (1/2390 [0.04%]) compared to 2018 (461/2583 [17.8%]). Further the newly implemented diagnostic strategy led to an increase (p = 0.08) in RSV infection rates in 2019 (111/2403 [4.6%]) compared to season 2018 (10/391 [2.6%]).

### Descriptive analysis of RSV patients

In total 103 patients with RSV infection were included into the analysis. Baseline characteristics are highlighted in Table 1. The median age of our cohort was 57 years and female and male patients were equally affected. Most of the patients suffered from comorbidities, such as cardiac illness (n = 54), pulmonary illness (n = 33), oncological disease (n = 24), type 2 diabetes mellitus (n = 20) and terminal dialysis-dependent kidney insufficiency (n = 13). 19 patients were solid-organ transplant recipients. Nearly half of the cohort (43.7%) required in-hospital care. Risk factors for hospitalization in RSV patients were, advanced age (> 65a) (p < 0.001), smokers (p < 0.001), comorbidities (p < 0.001), clinical or radiological signs for pneumonia (p = 0.009) and signs for superinfection (0.014) (supplementary Table 2). Inpatients had significant higher CRP values then patients outpatients (inpatients 1.3 mg/dl [0.52–2.91], outpatients 5.25 mg/dl [1.22–11.81]; p = 0.001) (supplementary table 3). Complications during hospitalization were most frequently worsening of pulmonary disease, such as COPD exacerbation in 16 patients, followed by cardiac decompensation in two patients. Superinfections were reported in 5 patients. *Streptococcus pneumoniae* detected by rapid urine antigen testing was the most common pathogen, followed by *Klebsiella pneumoniae* detected in blood culture (n = 1), *Enterococcus faecalis* detected in blood culture (n = 1) and *Aspergillus fumigatus* detected in a bronchoalveolar lavage fluid culture (n = 1).

**Table 1:** Demonstrates the baseline demographics of RSV patients.

Characteristics	RSV patients (n = 103)
Age—year median (Q1-Q3)	57 (40–73)
Female sex—no. (%)	54 (52.4)
BMI median (Q1–Q3)	25.3 (21–31.1)
Smoking—no. (%)	32 (31.1)
**Comorbidities—no. (%)**	
Respiratory illness	33 (32)
Cardiac illness	54 (52.4)
Type 2 diabetes mellitus	20 (19.4)
Dialysis	13 (12.6)
Oncological disease	24 (23.3)
SOT	19 (18.4)
Clinical or radiological findings for pneumonia—no. (%)	18 (17.5)
Respiratory complications—no. (%)	20 (19.4)
Cardiac complications—no. (%)	2 (1.9)
**Superinfections——no. (%)**	
Positive blood culture	2 (1.9)
Rapid urine antigen testing for *S. pneumoniae*	3 (2.9)
Positive broncho-alveolar lavage fluid culture	1 (1)
Antibiotics administered	29 (28.2)
Hospital admission	45 (43.7)
ICU admission—no. (%)	7 (6.8)
Length of stay—median (Q1–Q3)	13 (4–23)
Death—no. (%)	3 (2.9)

BMI, body-mass-index; SOT, solid-organ transplantation; ICU, intensive care unit.

**Figure 3:**
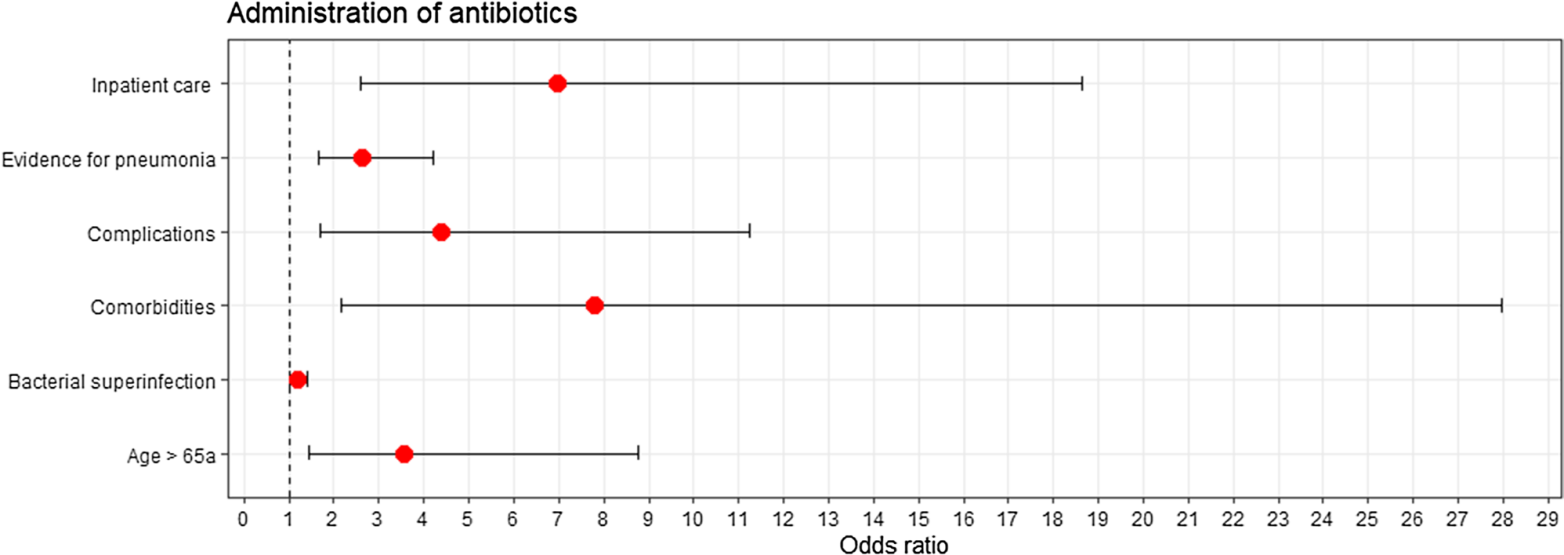
Demonstrates factors influencing administration of antibiotics in patients with RSV infections^20^. Listed factors were inpatient care (yes/no), evidence for pneumonia (defined as radiological evidence for pneumonia), complications (respiratory complications and bacterial or fungal superinfections), comorbidities (cardiac disease, pulmonary disease, type 2 diabetes mellitus, oncological disease, terminal dialysis-dependent kidney insufficiency and solid-organ transplant recipients), bacterial superinfections (bacterial growth in blood cultures) and age > 65a (yes/no).

Antimicrobial treatment was initiated in 29 of 103 (28.2%) patients. Decision for antimicrobial treatment was based on several factors as demonstrated in Fig. 3. Further, antimicrobial treatment was more likely to administered in patients with higher CRP values (no antibiotics prescribed CRP = 1.28 mg/dl [0.52–2.9], antibiotics prescribed CRP = 9 mg/dl [4.56–17.3]; p < 0.001]).

Of the patients admitted for in-patient care 15.56% (7/45) needed further treatment and an ICU ward. Patients with preexisting pulmonary (p = 0.03), complications of RSV infection (p = 0.001), pneumonia (p = 0.002) and bacterial superinfection (p = 0.036) were more likely to be admitted to ICU. Overall 2.9% of the patients died. Age over 65 was associated with higher odds for death. Table 2 demonstrated odds for hospitalization, ICU admission and mortality in dependence of several factors, such as age, smoking status, cardiac disease, pulmonary disease, type 2 diabetes mellitus, dialysis, oncological disease, solid-organ transplant recipient, complications aside RSV, pneumonia and superinfections.

**Table 2:** Highlights odds-ratios for different variables depending on outcome parameters hospitalization, ICU admission and mortality.

	Hospitalization		ICU admission		Mortality	
	OR (95% CI)	p-value	OR (95% CI)	p-value	OR (95% CI)	p-value
Age > 65 a	5.25 (2.2–12.5)	< 0.001	2.43 (0.51–11.51)	0.42	1.13 (1–12.74)	0.048
Smoking—yes	9.11 (3.4–24.38)	< 0.001	1.73 (0.36–8.24)	0.67	1.11 (0.1–12.74)	1
Cardiac illness	4.03 (1.75–9.28)	0.001	6 (0.7–51.74)	0.115	1.85 (0.16–21.02)	1
Respiratory illness	3.35 (1.41–7.96)	0.006	6.07 (1.11–33.16)	0.033	4.45 (0.39–50.95)	0.24
DM type II	2.96 (1.07–8.21)	0.044	1.73 (0.31–9.66)	0.62	9.11 (0.78–106.01)	0.096
Dialysis	3.38 (0.97–11.79)	0.07	0.87 (0.8–0.94)	0.59	0.87 (0.81–0.94)	1
Oncological illness	2.17 (0.86–5.49)	0.108	2.68 (0.56–12.92)	0.349	7.09 (0.61–81.9)	0.135
SOT	1.82 (0.66–5.02)	0.31	0.68 (0.08–5.97)	1	0.8 (0.72–0.89)	1
Complications	–	–	21.43 (2.44–187.98)	0.001	6 (0.52–69.03)	0.167
Pneumonia	4.3 (1.4–13.21)	0.009	15.96 (2.8–91.02)	0.002	10.5 (0.9–122.81)	0.078
Superinfection	–	–	12.4 (1.67–91.87)	0.036	12 (0.89–161.65)	0.14

SOT, solid-organ transplantation; ICU, intensive care unit.

### Assessment of the impact of clinicians' knowledge of RSV status on clinical practice

Finally, we reassessed 191 samples respiratory samples, negatively tested for influenza during RSV high peak during season 2018 but not tested for RSV. Indeed, we revealed 10 additional RSV cases. Handling of those ten patients (patients with unknown RSV infection) was compared to the handling of all other RSV patients (patients with known RSV infection). We did not detect differences in rates of hospitalization (patients with known RSV infection = 45/103 [43.7%], patients with unknown RSV infection = 4/10 [40%], p = 1.000) or frequency of antimicrobial administration (patients with known RSV infection = 29/103, 28.2%, patients with unknown RSV infection = 2/8, 20%, p = 0.725).

## Discussion

This study was conducted to assess the influence of a newly implemented combined flu/RSV test at the biggest tertiary care center in Austria. As expected, introduction of the test revealed that RSV is responsible for a substantial fraction of severe respiratory illnesses in adults during influenza season. Mortality in our cohort was high, and was even higher in patients with advanced age. Despite early and broad testing of our patients we were not able to safely discern any influence on admission rates to hospital or administration of antimicrobial agents.

Previously published studies demonstrated a particularly high disease burden of RSV disease burden in patients aged > 75years^6,21^. Consistent with previous reports, our patients were mostly of advanced age and suffered from chronical diseases, such as respiratory illness, cardiac illness, type 2 diabetes mellitus, oncological diseases or were solid-organ transplant recipients. Previous reports have demonstrated rates of bacterial superinfections in up to 15% of RSV cases^7^. In our cohort 11.1% of the patients suffered from superinfection. Most frequently reported pathogens were *Streptococcus pneumoniae*, *Klebsiella pneumoniae*, *Enterococcus faecalis* and *Aspergillus fumigatus*. Bacterial superinfections were associated with increased need for hospitalization and need for ICU admission.

Despite an increasing number of reports highlighting the disease burden of RSV in adults, awareness of many health care providers to adults seems to be low^12,13^. The clinical presentation of patients with RSV and influenza is non-specific and similar, which makes it almost impossible to differentiate between the viruses based on symptoms alone. Furthermore, RSV and influenza virus A and B epidemics show similar seasonality with most synchronized peaks in temperate regions. Chronological sequence of the viruses’ peak incidence differs depending on the country and is not necessarily consistent from year-to-year^22^. In Austria, the RSV epidemic starts later than influenza virus A^15,16^. As a result, clinicians in emergency rooms and ambulances had to separate patients with respiratory tract infections due to RSV and influenza quickly and reliably to prevent nosocomial transmission. We demonstrate that a combined test led to an expected increase in performed tests and detected cases, but also to an increase in detection rate from 2.6 to 4.6%. We believe that the demonstrated increase in detection rate emphasizes these diagnostic difficulties and supports the application of combined PCR tests in the clinical practice.

Broad testing did change the management of patients. Positively tested patients were admitted to distinct wards and had to remain in quarantine for 5 days. Further, health care workers in contact with those patients had to wear personal protective equipment. Herewith we achieved a save and controlled handling of RSV patients. Although, the implemented strategies suggest a reduction of nosocomial infection, the study design does not allow causative conclusions. Apart from the implemented measures, the impact of the test on the clinical reasoning is difficult to quantify. In theory, detection of viral pathogens responsible for pneumonia should reduce administration of antimicrobial treatment and prevent nosocomial infections. Coinciding data from previously published retrospective studies showed that increased viral testing only partially altered antimicrobial treatment^23,24^. Here, we demonstrate that reasoning for ab treatment depends on multiple factors such as an elevated CRP, clinical and radiological indication for pneumonia, as well as age of the patient rather than on the clinicians’ knowledge about RSV/test result.

Due to increasing availability and potency of viral diagnostic tools, viruses are increasingly detected as a cause for pneumonia world-wide. Clinical influence of broad viral testing was often discussed controversially, mostly due to three factors. Firstly, apart from neuraminidase inhibitors for influenza virus infections no specific antiviral options exists^25^. Secondly, attempts to provide convincing evidence that broad antiviral testing reduces rates of antimicrobial treatment were unsuccessful^23,24^. Thirdly, interpretation of naso-pharyngeal swabs poses a challenge of its own as the detected virus could be responsible for co-existing upper respiratory tract colonization or an actual pneumonia pathogen, making diagnosis difficult^25^. The above points should not be interpreted in the sense that testing is unnecessary, but rather that further research is urgently necessary to improve diagnostic procedures and the availability of treatment options. Currently, however, the biggest argument for broad-based testing remains the prevention of the spread of disease and protection of HCWs.

We acknowledge several limitations. First, our control group of patients with unknown RSV status was relatively small, as we only detected 10 additional cases. Hence, we only compared 10 patients from season 2018 to 103 cases in season 2019 in terms of clinical practice and reasoning, which is a limiting factor of our study. However, it is plausible clinicians’ select antimicrobial treatment based on multiple factors such as CRP, indication for pneumonia, age and not only on a test result. Furthermore, the assessed 191 samples corresponded to all samples during the suspected peak of RSV season in 2018, week 8 to 12. Hence, analysis of other periods would have been associated with increased efforts regrading costs and time. Secondly, we did not broadly test patients after the isolation period and cannot ultimately conclude if implemented strategies proved feasible in reducing risk of transmission after the isolation period. Thirdly, the present study was conducted retrospectively. Hence, study results were depended on the quality of documentation done by clinicians and does not allow conclusion about consequences not documented as, such as source of infection (nosocomial versus community acquired) and quality of care performed.

In conclusion, we demonstrated that although RSV disease is a well described cause for pneumonia in adults. Implementation of a combined influenza/RSV test led to a significant increase in detection rate, supported clinicians establishing the correct diagnosis and allowed a safe and controlled handling of RSV patients.

## Supplementary Information

Supplementary Information.

## Data Availability

The datasets generated during and/or analyzed during the current study are available from the corresponding author on reasonable request.

## References

[1] Nair H (2010). Global burden of acute lower respiratory infections due to respiratory syncytial virus in young children: A systematic review and meta-analysis. Lancet.

[2] Belongia, E. A. *et al.* Clinical features, severity, and incidence of rsv illness during 12 consecutive seasons in a community cohort of adults ≥ 60 years old. In *Open Forum Infect. Dis.* Vol. 5, (2018). 10.1093/ofid/ofy316 PMC6306566 30619907

[3] Kestler M, Muñoz P, Mateos M, Adrados D, Bouza E (2018). Respiratory syncytial virus burden among adults during flu season: An underestimated pathology. J. Hosp. Infect..

[4] Shi T (2020). Global disease burden estimates of respiratory syncytial virus-associated acute respiratory infection in older adults in 2015: A systematic review and meta-analysis. J. Infect. Dis..

[5] Topoulos S (2019). Analysis of acute respiratory infections due to influenza virus A, B and RSV during an influenza epidemic 2018. Infection.

[6] Matias G (2017). Estimates of hospitalization attributable to influenza and RSV in the US during 1997–2009, by age and risk status. BMC Public Health.

[7] Lee N (2013). High morbidity and mortality in adults hospitalized for respiratory syncytial virus infections. Clin. Infect. Dis..

[8] Malosh RE (2017). Respiratory syncytial virus hospitalization in middle-aged and older adults. J. Clin. Virol..

[9] Midgley CM (2017). Determining the seasonality of respiratory syncytial virus in the United States: The impact of increased molecular testing. J. Infect. Dis..

[10] Hall CB (1990). Occurrence of groups A and B of Respiratory syncytial virus over 15 years: Associated epidemiologic and clinical characteristics in hospitalized and ambulatory children. J. Infect. Dis..

[11] Hall CB, Douglas RG, Schnabel KC, Geiman JM (1981). Infectivity of respiratory syncytial virus by various routes of inoculation. Infect. Immun..

[12] Thompson WW (2003). Mortality associated with influenza and respiratory syncytial virus in the United States. JAMA.

[13] Ackerson B (2019). Severe morbidity and mortality associated with respiratory syncytial virus versus influenza infection in hospitalized older adults. Clin. Infect. Dis..

[14] Benchimol EI (2015). The REporting of studies Conducted using Observational Routinely-collected health Data (RECORD) Statement. PLoS Med..

[15] Monika Redlberger-Fritz, T. P.-K. Austria’s RSV Sentinel Physician Surveillance Network. 10.1371/journal.pone.0149916 PMC4790898 26975056

[16] Austria’s Influenza Sentinel Physician Surveillance Network.

[17] Ward C (2004). Design and performance testing of quantitative real time PCR assays for influenza A and B viral load measurement. J. Clin. Virol..

[18] Fry AM (2010). The Burden of Hospitalized Lower Respiratory Tract Infection due to Respiratory Syncytial Virus in Rural Thailand. PLoS ONE.

[19] R Core Team. R: A language and environment for statistical computing. http//www.R-project.org/ (R Found. Stat. Comput., Vienna, 2014).

[20] Wickham H. ggplot2: Elegant Graphics for Data Analysis. ISBN 978-3-319-24277-4, https://ggplot2.tidyverse.org (Springer, New York, 2016).

[21] Zhou H (2012). Hospitalizations associated with influenza and respiratory syncytial virus in the United States, 1993–2008. Clin. Infect. Dis..

[22] Lam TT (2019). Comparative global epidemiology of influenza, respiratory syncytial and parainfluenza viruses, 2010–2015. J. Infect..

[23] Akers IE (2017). Influence of time to diagnosis of severe influenza on antibiotic use, length of stay, isolation precautions, and mortality: A retrospective study. Influenza Other Respir. Viruses.

[24] Walter JM, Wunderink RG (2018). Testing for respiratory viruses in adults with severe lower respiratory infection. Chest.

[25] Ruuskanen O, Lahti E, Jennings LC, Murdoch DR (2011). Viral pneumonia. Lancet.

