# Supplementary material for: Surveillance of respiratory syncytial virus infections in adults, Austria, 2017 to 2019
Source: Sci Rep. 2021 Apr 26;11:8939. doi: 10.1038/s41598-021-88537-5 (PMC8076173; doi:10.1038/s41598-021-88537-5)
Supplement: Supplementary file 1 — Supplementary Information. [file 41598_2021_88537_MOESM1_ESM.docx]

Supplementary data:

**Surveillance of respiratory syncytial virus infections in adults, Austria, 2017 to 2019**

Supplementary table 1 demonstrates implemented infection control and prevention strategies for the handling of RSV and influenza virus patients.

| Implemented infection control and prevention strategies |
| --- |
| 1. All patients reporting respiratory symptoms and admitted to the hospital, within the influenza and RSV season, were tested for influenza virus A, B and RSV |
| 2. All RSV positively tested patients in need for in-hospital care were admitted to distinct wards to maintain isolation for 5 days |
| 3. Personal protective equipment (consisting of FFP2 masks [FFP3 for aerosol generated procedures], goggles or face shields, long-sleeved water resistant gowns and a minimum of one pair of gloves) for HCWs in contact with RSV patients |
| 4. Hand hygiene according to recommendations of the WHO ^26^ |
| 5. All HCWs at the center were offered free influenza vaccination |

HCWs, Health Care Workers; FFP, Filtering Face Piece; WHO, World Health Organization

Supplementary table 2. Comparison of the baseline demographics in dependence of hospitalization.

| **Characteristics** | | **Non-hospitalized patients (n= 58)** | **Hospitalized patients (n= 45)** | **p-value** |
| --- | --- | --- | --- | --- |
| Age – yr median (Q1-Q3) | | 50 (34 – 62) | 70 (57 – 77) | <0.001 |
| Female sex – no. (%) | | 29 (50) | 25 (55.6) | 0.69 |
| BMI median (Q1-Q3) | | 26.7 (19.8 – 35.5) | 24.1 (21.4 – 27.3) | 0.38 |
| Smoking – no. (%) | | 7 (12.1) | 25 (55.6) | <0.001 |
| Comorbidities – no. (%) | | 25 (43.1) | 40 (88.9) | <0.001 |
|  | Respiratory illness | 12 (20.7) | 21 (46.7) |  |
|  | Cardiac illness | 22 (37.9) | 32 (71.1) |  |
|  | T2DM | 7 (12.1) | 13 (28.9) |  |
|  | Dialysis | 4 (6.9) | 9 (20) |  |
|  | Oncological disease | 10 (17.2) | 14 (31.1) |  |
|  | SOT | 8 (13.8) | 11 (24.4) |  |
| Radiological findings for pneumonia – no.(%) | | 5 (8.6) | 13 (28.9) | 0.009 |
| Superinfection – no.(%) | | 0 | 5 (11.1) | 0.014 |

T2DM, type 2 diabetes mellitus; SOT, solid-organ transplantation

Supplementary table 3 demonstrates levels of laboratory parameters in dependence of hospitalization and administration of antibiotics.

| Laboratory parameters (median – Q1-Q3) | Hospitalization | | | Administration of antibiotics | | |
| --- | --- | --- | --- | --- | --- | --- |
|  | no | yes | p-value | no | yes | p-value |
| Hemoglobin (g/dl) | 12.6 (11.9 -14.3) | 11.6 (10.1 – 13.5) | 0.025 | 12.4 (11.5 – 14.1) | 11.9 (10.4 – 13.8) | 0.36 |
| Thrombocytes (G/L) | 208 (182 – 243) | 225 (169 – 278) | 0.7 | 209 (174 – 250) | 232 (178 – 260) | 0.83 |
| Leucocytes (G/L) | 4.87 (8.75 – 8.16) | 8.16 (5.8 – 11.16) | 0.12 | 7.07 (5.19 – 8.79) | 8.58 (6.19 – 11.05) | 0.13 |
| Creatinine (mg/dl) | 0.98 (0.2 – 0.6) | 1.02 (0.74 – 1.69) | 0.48 | 0.96 (0.75 – 1.31) | 1.02 (0.86 – 1.66) | 0.29 |
| Bilirubin (mg/dl) | 0.38 (0.2 – 0.6) | 0.41 (0.29 - 0.63) | 0.45 | 0.4 (0.24 – 0.62) | 0.41 (0.21 – 0.73) | 0.79 |
| CRP (mg/dl) | 1.3 (0.52 – 2.91) | 5.25 (1.22 – 11.81) | 0.001 | 1.28 (0.52 – 2.56) | 9 (4.56 – 17.3) | <0.001 |
